# Bone Mineral Density During and After Lactation: A Comparison of African American and Caucasian Women

**DOI:** 10.1007/s00223-023-01125-9

**Published:** 2023-08-28

**Authors:** Marilyn Augustine, Robert Boudreau, Jane A. Cauley, Deborah Majchel, Nayana Nagaraj, Lauren S. Roe, Poonam Sood, Andrew F. Stewart, Mara J. Horwitz

**Affiliations:** 1 University of Rochester School of Medicine, Rochester, NY USA; 2 University of Pittsburgh School of Public Health, Pittsburgh, PA USA; 3 Intercostal Medical Group, Sarasota, FL USA; 4 Lyndra Therapeutics Inc, Watertown, MA USA; 5 Atrium Health, Charlotte, NC USA; 6 Icahn School of Medicine at Mount Sinai, New York, NY USA; 7 University of Pittsburgh School of Medicine, Pittsburgh, PA USA

**Keywords:** DXA, Biochemical markers of bone turnover, Systems biology, Lactation, Race/ethnicity(other), Parathyroid hormone

## Abstract

During lactation, changes in maternal calcium metabolism are necessary to provide adequate calcium for newborn skeletal development. The calcium in milk is derived from the maternal skeleton through a process thought to be mediated by the actions of parathyroid hormone-related protein (PTHrP) in combination with decreased circulating estrogen concentrations. After weaning, bone lost during lactation is rapidly regained. Most studies of bone metabolism in lactating women have been performed in Caucasian subjects. There are well-documented differences between African American (AA) and Caucasian (C) bone metabolism, including higher bone mineral density (BMD), lower risk of fracture, lower 25-hydroxyvitamin D (25(OH) D), and higher PTH in AA compared to C. In this prospective paired cohort study, BMD and markers of bone turnover were compared in self-identified AA and C mothers during lactation and after weaning. BMD decreased in both AA and C women during lactation, with similar decreases at the lumbar spine (LS) and greater bone loss in the C group at the femoral neck (FN) and total hip (TH), demonstrating that AA are not resistant to PTHrP during lactation. BMD recovery compared to the 2 week postpartum baseline was observed 6 months after weaning, though the C group did not have complete recovery at the FN. Increases in markers of bone formation and resorption during lactation were similar in AA and C. C-terminal telopeptide (CTX) decreased to 30% below post-pregnancy baseline in both groups 6 months after weaning, while procollagen type 1 N-terminal (P1NP) returned to baseline in the AA group and fell to below baseline in the C group. Further investigation is required to determine impacts on long term bone health for women who do not fully recover BMD before a subsequent pregnancy.

## Introduction

Alterations in calcium and bone metabolism occur during lactation to provide adequate calcium for the skeletal development of the infant. During lactation, mothers who are exclusively breastfeeding lose 200 mg of calcium or more each day in breast milk [1–3]. Although increased intestinal calcium absorption is observed during pregnancy, the main mechanism by which lactating mothers provide adequate breast milk calcium for newborn skeletal development appears to be maternal bone resorption.

Decreases in areal bone density of 2–8% have been observed in several longitudinal studies over the first 2–6 months of lactation [4–8]. Additional studies have revealed changes in bone microstructure in lactating women [9, 10]. Most studies have found markers of bone formation and resorption to be elevated during lactation beyond levels found in the third trimester of pregnancy [11–14]. The bone resorption observed in lactation is largely mediated by parathyroid hormone related protein (PTHrP) in the setting of low circulating estrogen concentrations [15–17]. The maternal bone lost during pregnancy and lactation appears to be at least partially regained after weaning [18], and trabecular bone starts to be regained even during prolonged breastfeeding [19, 20]. Data regarding the rate of bone loss during lactation and time to recovery after weaning remains limited.

Most studies of bone metabolism during lactation have been performed in Caucasian or Asian populations. However, there are well-documented differences in bone metabolism between African American (AA) and Caucasian (C) women. AA women tend to have lower 25-hydroxyvitamin D (25OHD) and higher circulating parathyroid hormone (PTH) concentrations compared to C women [21]. AA women also have lower levels of vitamin-D binding protein compared to C women, so that despite lower levels of total 25-hydroxyvitamin D, levels of bioavailable 25-hydroxyvitamin D are similar [22]. Furthermore, there is evidence that AA people have lower skeletal sensitivity to the resorptive effects of PTH, which could help to explain the higher bone mineral density (BMD) and lower risk of fracture that has been observed in AA as compared to C women [23, 24]. These findings raise the question of whether the AA skeleton might be resistant to the effects of PTHrP in lactation. Studies of lactation-associated bone loss in Black Gambian women have demonstrated similar lactation-associated losses in BMD between Gambian women and a comparison group of Caucasian women in the UK [25–27], suggesting that these losses occur regardless of race. However, the groups differed significantly in terms of intake of calcium and other nutrients.

Our group previously showed similar increases in serum markers of bone turnover in AA and C mothers in a brief 12-week lactation study [28]. Questions remain as to how bone turnover markers in AA vs. C compare over a longer period of lactation and whether changes in bone density during lactation and weaning differ between groups with similar nutrient intake. Here we present what we believe is the first study to assess longitudinal changes in BMD and bone turnover markers over a more prolonged period of lactation in AA compared to C mothers.

## Materials and Methods

### Study Subjects

Subjects included healthy AA and C mothers ages 21–45 who were postpartum after a singleton pregnancy. Race was self-reported. All participants were breastfeeding, defined as exclusively breastfeeding or using up to one bottle of supplemental formula per day, at the time of the first two study visits.

Exclusion criteria included disorders which could affect bone metabolism, including renal, cardiovascular, pulmonary, malignant, hepatic, hematologic or rheumatologic disease. Other exclusion criteria included fractures or bone surgery within the past 12 months, smoking, history of significant alcohol or drug use, chronic medications including depot medroxyprogesterone acetate (DMPA) (with the exceptions of stable doses of thyroid hormone or vitamin supplements), weight over 130 kg, and BMD Z-score of − 3.0 or lower at the hip or spine. Women who achieved pregnancies with in vitro fertilization (IVF) or other hormonal manipulation were excluded, as were those who had significant complications with the most recent pregnancy or were unable to exclusively breastfeed beginning at birth. Women who became pregnant during the study did not participate in study visits after pregnancy was discovered. The study protocol was approved by the University of Pittsburgh Institutional Review Board and informed consent was obtained from each subject prior to the initiation of study procedures. The study was performed in accordance with the ethical standards and principals of the Declaration of Helsinki.

### Study Design

This was a prospective paired cohort study comparing bone mineral density (BMD) and markers of turnover during lactation and weaning in self-identified AA and C mothers. The primary endpoint was BMD at the lumbar spine (LS), total hip (TH) and femoral neck (FN). Based on reported BMD changes and our prior bone marker results in C women [11], we planned to recruit 25 subjects per group with the goal of at least 20 women per group completing the study.

The study involved four outpatient visits to the Endocrine & Metabolism Research Center at the University of Pittsburgh. The first visit occurred two weeks after delivery (baseline), followed by visits at 12 weeks postpartum (3 months), at 24 weeks postpartum or at the onset of weaning if prior to 24 weeks (6 months), and then 6 months after completely weaning (post-wean). At each visit, a relevant medical history, calcium and physical activity questionnaires, blood and urine samples, breast milk samples, and a dual energy x-ray absorptiometry (DXA) scan of the LS, TH, FN and distal 1/3 of the radius (DR) of the non-dominant limb were obtained. Blood samples were collected for calcium, ionized calcium, albumin, phosphate, PTH (1–84), 25-hydroxyvitamin D (25-OHD), estradiol, and markers of bone turnover [procollagen type 1 N-terminal (P1NP) and C-terminal telopeptide (CTX)] at each visit. Urine calcium, creatinine, and phosphate were measured at every visit on a second morning void. TSH was measured at the 3 month visit. Only subjects who completed both the baseline and 3 month visits and who were still breast feeding at three months were included in the final analysis.

### Laboratory Assays and DXA

Serum total calcium, ionized calcium, albumin, phosphate, and TSH were measured using standard automated chemistries in the University of Pittsburgh Medical Center (UPMC) Clinical Chemistry Laboratory on the day of collection. Markers of bone turnover, 25-OH D, estradiol and intact PTH were assayed in single batches at the conclusion of the study on blood samples that had been processed and stored at − 80 °C. 25-OH D was measured in the UPMC Clinical Chemistry Laboratory by Liquid Chromatography-Tandem Mass Spectroscopy using Waters Oasis HLB SPE columns (Waters Corporation, United Kingdom, CV = 10%). Intact PTH (1–84) was measured by immunochemiluminometric assay (Quest Laboratories, San Juan Capistrano, CA). P1NP was measured using the IDS-iSYS Intact PINP amino-terminal propeptide of type I procollagen assay (Immunodiagnostics Systems, United Kingdom, CV = 2.2%) and CTX was measured using the Serum Crosslaps ELISA assay (Immunodiagnostics Systems, United Kingdom, CV < 11%). A urine pregnancy test was obtained at each visit prior to the DXA scan (ICON urine hCG).

Spot urine calcium, creatinine and phosphate were measured on the day of each visit using standard automated chemistries in the UPMC Clinical Chemistry Laboratory. Fasting samples were collected when possible, but many patients found it difficult to fast while breastfeeding, so some samples were non-fasting. Fractional excretion of calcium was calculated using FECa = (urine calcium/ionized serum calcium)/(urine creatinine/serum creatinine). Tubular maximum reabsorption of phosphate (TMP) was calculated as described by Payne [29].

DXA scans were all performed by trained study personnel using the same Lunar iDXA machine (GE Healthcare) (in house CV = 1.48%). A single investigator reviewed all scans for comparison to baseline. Calibration of the absorptiometers was done before each study visit using a Lunar phantom. Breasts were shielded for scans done at visits when subjects were lactating.

### Statistics

Baseline characteristics of participants were reported by race as mean (SD) or median (25th percentile, 75th percentile) depending on normality. Comparisons of characteristics between races were conducted using equal or unequal variance t-tests, or a Wilcoxon Rank Sum test as indicated. With close to 50% of the sample having their first child, the distribution of parity in each race exhibited a distinct peak at parity = 1, then tapered off at higher parity values. Subtracting one from parity values, the distributions of parity-1 between races was compared using a negative binomial distribution. This distribution fit better than a Poisson distribution based on AIC.

The primary focus was on changes from baseline when comparing trends over time in BMD. For absolute changes from baseline to 3 months postpartum, 6 months postpartum and 6 months post wean, 4-timepoint longitudinal repeated measures mixed models were used with baseline as the referent. To reduce the number of covariance parameters estimated so that means are better estimated, we conducted goodness-of-fit test using the AIC criterion to identify best-fitting parsimonious covariance structures for each measure and each race. Covariance structures compared were: unstructured, auto-regressive with equal variances at each timepoint, heterogeneous auto-regressive, equi-correlated with equal-variances and heterogenous equi-correlated. Percent changes from baseline were also examined. Percent changes at 3 months, 6 months and 6 months post wean were modeled using 3-timepoint longitudinal repeated measures mixed models with identification of best fitting parsimonious covariance structures using a similar approach. The final repeated measures models included race by timepoint interactions which provided statistical tests for differences in change from baseline at each timepoint between races. For BMD measures over time, changes vs. baseline plus 95% CIs were tabulated at the three post-baseline timepoints. Similar measures were used for analysis of the biomarkers as a secondary endpoint. However, the time-point specific values plus 95% CIs, rather than the changes, were tabulated at each of the post-baseline timepoints. In both cases, the p-values evaluating differences by race at each timepoint are for changes vs baseline based on the relevant repeated measures model. All analyses were conducted using SAS version 9.4 (SAS Institute Inc., Cary). Box plots were created using R version 4.1.2 (2021-11-01).

## Results

### Demographics

**Fig. 1:**
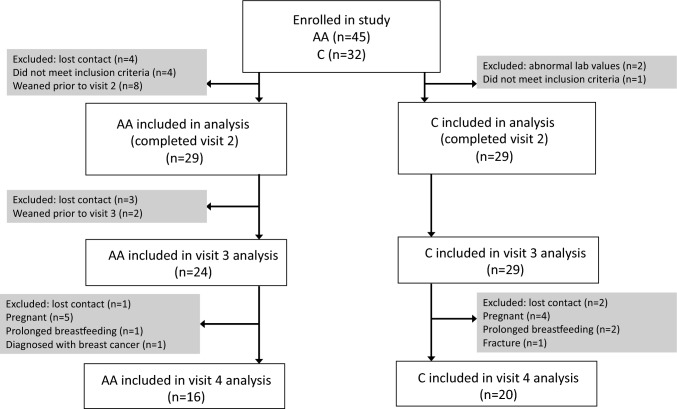
Enrollment flowsheet

Forty-five AA and 32 C subjects were enrolled. Of the 29 subjects in each group who completed visits 1 and 2 and were included in the analysis, 16 AA subjects and 20 C subjects completed all four study visits (Fig. 1).

Review of EMR data during the 14-month recruitment period (10/2012–12/2013) indicates that there were 12,230 deliveries at UPMC Magee, a women’s health and maternity hospital from which most subjects were recruited. Of these, 2402 mothers self-identified as AA and 230 did not specify their race. Recruitment of AA participants was considerably more challenging than recruitment of C participants. Although a majority of mothers were breastfeeding at the time of discharge, a smaller percentage for AA new mothers (52%, n = 1252) as compared to non-AA new mothers (74%, n = 7093) were breastfeeding. Furthermore, 638 (27%) AA new mothers received DMPA for birth control prior to discharge, and were therefore not eligible for this study, as compared to 494 (5%) of non-AA new mothers.

AA and C lactating mothers were similar at baseline (2 weeks postpartum) with regard to parity, number of breastfeeding sessions per day, and amount of physical activity per day, as shown in Table 1. AA subjects had a significantly higher BMI than C subjects throughout the study. p values for change in weight over time were nonsignificant for both groups; however, AA subjects’ weight trended up and C subjects’ weight trended down over the study. Baseline average total daily calcium intake through food and supplements was significantly lower in AA mothers than C mothers (955 mg/day vs 1282 mg/day, p = 0.03). Total daily calcium intake decreased in both groups by the final visit, with AA subjects’ calcium intake remaining lower than that of C subjects for the entire study duration.

**Table 1:** Baseline characteristics

	African American (n = 29)	Caucasian (n = 29)	P value
Age (years)	30.52 (6.19)	31.86 (4.07)	0.33
Parity	2.62 (1.93) 1st child: 38%, range:1–9	1.97 (1.30) 1st child: 48%, range:1–5	0.13^#^
Breast feeding sessions per day	9.76 (2.05)	10.79 (2.81)	0.12
Weight (kg)	83.62 (17.81)	71.73 (10.61)	**0.003**
BMI (kg/m^2^)	30.79 (6.31)	26.49 (3.82)	**0.003**
SBP (mm Hg)	122.34 (16.06)	111.72 (10.13)	**0.004**
DBP (mm Hg)	75.90 (10.28)	70.24 (9.22)	**0.03**
Sitting time (min/day)	635.60 (398.82)	668.89 (248.89)	0.72
Total MET (min/week)	1895.93 (3083.35)	1108.26 (1602.1)	0.27
Dietary calcium (mg/day)	861.93 (455.91)	1063.9 (452.32)	0.10
Average total daily calcium (mg)	955.31 (458.63)	1281.62 (525.54)	**0.03**
Serum total calcium (mg/dl)	9.27 (0.33)	9.14 (0.33)	0.15
Ionized calcium (mg/dl)	4.66 (0.19)	4.67 (0.18)	0.92
Creatinine (mg/dl)	0.77 (0.13)	0.71 (0.13)	0.11
FeCa	0.01 (0.05)	0.02 (0.02)	**0.009**
Phosphate (mg/dl)	3.47 (0.45)	3.97 (0.59)	**0.0006**
TMP/GFR	3.86 (0.61)	4.15 (0.84)	0.13
Albumin (mg/dl)	3.76 (0.37)	3.65 (0.28)	0.24
Total 25-OH Vitamin D (ng/ml)	28.72 (11.15)	44.03 (13.07)	** < 0.0001**
PTH (pg/ml)	44.90 (30.12)	37.97 (29.74)	0.38
Estradiol (pg/ml)	31.10 (10.93)	18.28 (13.27)	**0.0002**
CTX (ng/ml)	0.66 (0.29)	0.55 (0.21)	0.10
P1NP (ng/ml)	84.09 (37.56)	77.80 (33.97)	0.51
LS BMD (g/cm^2^)	1.31 (0.16)	1.15 (0.12)	** < 0.0001**
Total Hip BMD (g/cm^2^)	1.12 (0.12)	1.00 (0.11)	**0.0003**
FN BMD (g/cm^2^)	1.14 (0.16)	1.00 (0.10)	**0.0005**
Distal 1/3 Radius BMD (g/cm^2^)	0.90 (0.06)	0.85 (0.07)	**0.004**

Bold values represent differences that are statistically different

^#^Negative binomial test for parity-1, all others—t-tests

For this study, breastfeeding was defined as exclusively breastfeeding or using up to one bottle of supplemental formula per day. Most mothers were not using any supplemental formula at baseline. At 3 months, 10 mothers in the AA group and 2 in the C group were using up to one bottle of supplemental formula per day. The study was designed to include women who were still breastfeeding at 6 months and subjects were instructed to contact the study team if they began to wean before 6 months so they could be brought in for the 3rd study visit before they began significant weaning. Despite this design, at the 6 month visit, 7 AA and 2 C mothers were using more than one supplemental bottle per day and one AA mother had fully weaned. By defininion, all subject had fully weaned 6 months before the last study visit. One subject in the AA group and two subjects in the C group could not complete the post wean visit as they were still breastfeeding, each for over 2 years, when the study was closed. Median duration of breastfeeding did not differ between groups [median (25^th^ percentile, 75th percentile): AA = 9.0 months (6.5, 11.0); C = 8.0 months (6.0, 12.5); p = 0.80].

All subjects had resumed menses at the time of weaning. On average menses resumed at 5 months postpartum for both groups (AA range 2–14 months, C range 1.5–18 months).

### Baseline Laboratory Analysis

At baseline, total 25-OH Vitamin D levels were lower in AA subjects than in C subjects (28.72 vs 44.03 ng/ml, p < 0.0001). PTH was marginally, but not significantly higher in AA subjects compared to C subjects at baseline. Serum phosphate was lower in AA subjects than in C subjects which corresponded with a trend for lower ratio of tubular maximum reabsorption of phosphate to glomerular filtration rate (TMP/GFR) in the AA group. There were no statistically significant differences between groups at baseline with regard to total calcium, ionized calcium, creatinine, or albumin. However, FECa was lower in AA participants as compared to C participants (Table 1).

Estradiol levels were significantly higher in AA women as compared to C women and this difference remained significant when adjusted for BMI. There were no statistically significant differences between groups in baseline markers of bone turnover as measured by CTX and P1NP (Table 1).

### Bone Mineral Density

At baseline AA participants had significantly higher absolute BMD at all sites compared to C participants (Table 1).

**Fig. 2:**
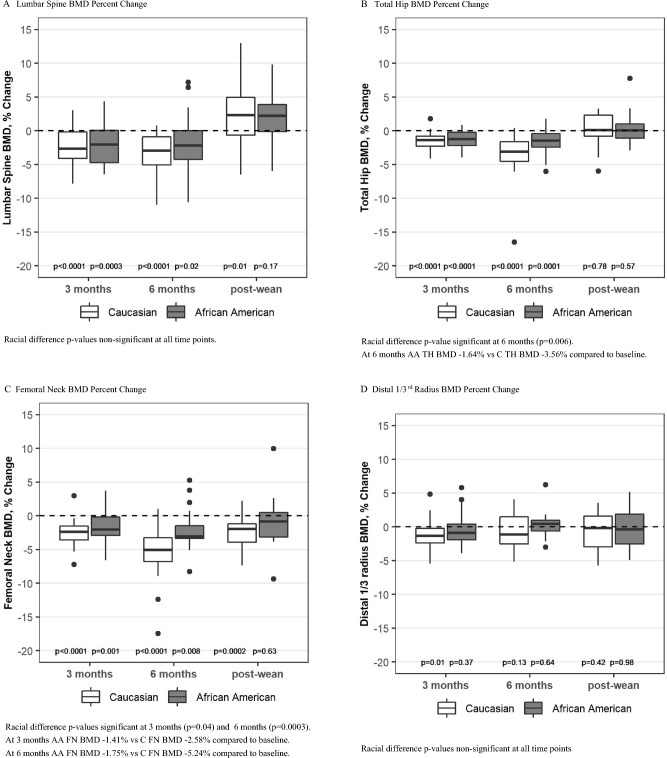
Percent change in BMD from baseline (unadjusted). Box plots show median, interquartile range, max and min values with outlying data points plotted. p-values on the figure are for change from baseline

AA Group: BMD decreased from baseline at the LS, TH, and FN in the AA group at 3 and 6 months and then returned to baseline at all three sites after weaning (Table 2, Fig. 2A–C). Distal radius (DR) BMD remained near baseline at all time points for the AA group (Table 2, Fig. 2D).

**Table 2:** Absolute change in BMD from baseline (gm/cm^2^) [unadjusted]

	3 month Mean (95% CI) p compared to baseline	6 month Mean (95% CI) p compared to baseline	6 months post-wean Mean (95% CI) p compared to baseline
African American	*n* = *29*	*n* = *24*	*n* = *16*
Caucasian	*n* = *29*	*n* = *29*	*n* = *20*
LS BMD			
African American	**− 0.03 (− 0.05,− 0.02) p = 0.0001**	**− 0.03 (− 0.05,− 0.01) p = 0.008**	0.01 (− 0.02, 0.04) p = 0.41
Caucasian	**− 0.03 (− 0.04,− 0.02) p < 0.0001**	**− 0.04 (− 0.05,− 0.02) p < 0.0001**	**0.03 (0.00, 0.05) p = 0.02**
FN BMD			
African American	**− 0.02 (− 0.03,− 0.01) p = 0.001**	**− 0.02 (− 0.03,− 0.01)**† **p = 0.0008**	0.005 (− 0.03, 0.04) p = 0.79
Caucasian	**− 0.03 (− 0.03,− 0.02) p < 0.0001**	**− 0.05 (− 0.07,− 0.04)**† **p < 0.0001**	**− 0.03 (− 0.04,− 0.01) p = 0.0006**
Total Hip BMD			
African American	**− 0.01 (− 0.02,− 0.01) p = 0.0001**	**− 0.02 (− 0.03,− 0.01)**† **p = 0.0008**	0.004 (− 0.01, 0.02) p = 0.53
Caucasian	**− 0.02 (− 0.02,− 0.01) p < 0.0001**	**− 0.04 (− 0.05,− 0.02)**† **p < 0.0001**	− 0.01 (− 0.02, 0.01) p = 0.49
Distal 1/3rd Radial BMD			
African American	− 0.004 (− 0.01, 0.005) p = 0.36	0.001 (− 0.01, 0.01) p = 0.82	− 0.002 (− 0.01, 0.01) p = 0.67
Caucasian	**− 0.01 (− 0.02,− 0.002) p = 0.02**	− 0.01 (− 0.01, 0.002) p = 0.14	− 0.005 (− 0.02, 0.01) p = 0.39

Bold values represent differences that are statistically different

^†^p value for racial difference in change significant for FN BMD at 6 months (p = 0.002) and TH BMD at 6 months (p = 0.02)

C Group: BMD also decreased from baseline at the LS, TH, and FN in the C group at 3 and 6 months and then returned to baseline at the LS and TH after weaning (Table 2, Fig. 2A, B). There was a persistent loss of BMD at the FN at 6 months post-wean in the C group (Fig. 2C). DR BMD decreased by 1.08% (p = 0.01) in the C group at 3 months (Table 2, Fig. 2D) and did not differ from baseline at 6 months and post-wean.

Racial Comparison: The decrease in BMD at the LS did not differ between the groups. However, at 6 months postpartum, the decrease in BMD at the TH was greater for the C group compared to the AA group (Table 2 and Fig. 2B). The FN was the site of greatest absolute bone loss in the C group with a 5.24% loss at 6 months postpartum (p < 0.0001) and a persistent loss of 2.64% at 6 months post-wean (p = 0.0002) (Fig. 2C). In contrast, FN BMD in the AA group was only 1.75% below baseline at 6 months postpartum (p = 0.007) and had returned to baseline by 6 months post wean (p = 0.66). The p-values were significant for racial differences at the FN at 3 months and 6 months (Table 2, Fig. 2C) and for change over time (p = 0.02).

BMI was positively associated with FN BMD (p = 0.0005), TH BMD (p = 0.001) and DR BMD (p = 0.001) but not significantly associated with LS BMD. Significance of racial differences in BMD did not change after adjustment for BMI.

### Changes in Hormones

Estradiol levels rose over time in both groups (Table 3). Despite higher baseline estradiol levels, there was also a significantly greater increase from baseline estradiol levels in AA women compared to C women 6 months post wean (178.53 vs 75.58 pg/ml, p = 0.03). Adjustment for BMI did not change significance of racial differences in estradiol levels.

**Table 3:** Laboratory values over time after baseline

	3 month Mean (95% CI) p compared to baseline	6 month Mean (95% CI) p compared to baseline	6 months ost-wean Mean (95% CI) p compared to baseline
African American	*n* = *29*	*n* = *24*	*n* = *16*
Caucasian	*n* = *29*	*n* = *29*	*n* = *20*
Estradiol (pg/ml)			
African American	**53.76 (40.26,67.26) p = 0.001**	**71.07 (39.63,102.51) p = 0.01**	**178.53 (102.30,254.76)**† **p = 0.0007**
Caucasian	**41.90 ( 22.29,61.51) p = 0.01**	**59.56 (31.78,87.34) p = 0.005**	**75.58 (53.57,97.59)**† **p < 0.0001**
PTH (pg/ml)			
African American	**55.24 ( 41.19, 69.29) p = 0.04**	47.97 (39.64,56.30) p = 0.45	50.96 (34.00,67.92) p = 0.25
Caucasian	36.83 (30.52, 44.14) p = 0.81	42.38 (34.08,50.68) p = 0.37	47.57 (37.21,57.93) p = 0.08
TMP/GFR			
African American	**3.53 (3.26, 3.80)** **p = 0.03**	3.65 (3.42, 3.88) p = 0.18	**3.30 (2.89, 3.71)** **p = 0.003**
Caucasian	4.11 (3.76, 4.46) p = 0.79	**3.69 (3.41, 3.97)** **p = 0.02**	**3.09 (2.78, 3.40) p < 0.0001**
Total 25-OH Vitamin D (ng/ml)			
African American	25.96 (22.51,29.41)† p = 0.09	**24.13 (20.32,27.94) p = 0.0386**	23.34 (17.72, 28.96) p = 0.05
Caucasian	**36.69 (32.82,40.56)**† **p < 0.0001**	**37.79 (34.30,41.28) p = 0.002**	**36.96 (29.08,44.84) p = 0.04**

Bold values represent differences that are statistically different

See Table 1 for baseline lab values

^†^p value for racial difference in change significant for Estradiol at 6 months post-wean (p = 0.03) and Total 25-OH vitamin D at 3 Months (p = 0.04)

Vitamin D levels decreased from baseline in both groups, though the difference between groups only met significance at the 3 month timepoint (p = 0.04). BMI was negatively associated with Total 25-OH Vitamin D (p = 0.003). Adjustment for BMI did not change significance of racial differences in vitamin D. Overall PTH was stable throughout all timepoints the study with no racial differences.

### Markers of Bone Turnover

**Fig. 3:**
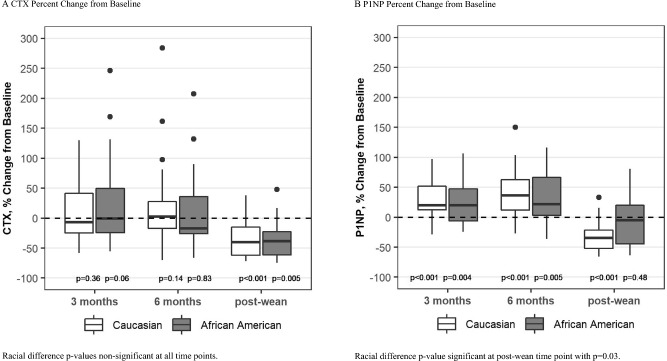
Box plots show median, interquartile range, max and min values with outlying data points plotted. p-values on the figure are for change from baseline

Bone resorption, as measured by CTX was stable during lactation and then decreased to 30% below baseline by 6 months after weaning in both groups (change from baseline p = 0.005 for AA and p < 0.0001 for C, Fig. 3A). There was no overall difference between the groups over time.

Bone formation, as measured by P1NP, increased significantly during lactation in both groups. After weaning, P1NP levels returned to baseline in the AA group but fell to 32% below baseline in the C group (p < 0.0001 for C, p = 0.03 for racial difference, Fig. 3B).

## Discussion

To our knowledge, this is the first study comparing changes in BMD and bone turnover during and after lactation in AA and C mothers. The baseline BMD results confirm the previously described racial differences in bone density [24], as BMD was higher in AA than C participants at all sites. The finding that BMD decreased in both AA and C women during the first 6 months of lactation is novel but not unexpected.

It is believed that much of the bone loss that occurs during lactation is mediated by breast derived PTHrP [2]. The fall in BMD in the AA participants in this study demonstrates that lactating AA mothers are not resistant to PTHrP and other hormones affecting bone metabolism during lactation despite data suggesting that AA women may be resistant to PTH [23]. However, it is of note that the decrease in BMD was slightly greater in the C group at the TH and FN, and that BMD did not completely recover compared to the 2 week postpartum baseline at the FN by 6 months after weaning in the C group despite the fact that all subjects had resumed menses prior to weaning. This is consistent with previous studies in C women which suggested that cortical bone-rich sites may take longer to recover from bone loss during lactation as compared to spine [18, 30]. Some studies [9, 10] found permanent skeletal deficits in at least some women who breastfed for nine months or more, but this has never been studied in AA women. Because of small sample size, we did not have the power to examine effect of duration of breastfeeding on bone loss or recovery. We hypothesize that the differences in bone loss between the AA and C groups may be in part be explained by differences in bone turnover as well as differences in estradiol as discussed further below.

CTX as a marker of bone resorption was stable during 6 months of lactation, then decreased to below the lactation values after weaning in both groups, as would be expected to permit recovery of BMD. CTX did not differ in the two groups in this study, whereas we reported a lower value in the lactating AA women in our previous study [28]. It is possible that the interracial comparison may have been significantly different if a larger sample size was used. It is important to note that this study used the 2 week postpartum value as the baseline and lacked pre-pregnancy or age and BMI matched non-pregnant controls. Based on the significant fall in CTX with weaning, we hypothesize that the CTX levels at the baseline (2 week postpartum) visit in this study represent an already high state of bone turnover compared to pre-pregnancy baseline.

In contrast to stable CTX levels during lactation, P1NP, as a marker of bone formation, increased significantly during lactation in both groups compared to baseline indicating that bone formation may have been slightly slower to turn on than bone resorption. Since BMD fell during this time, this paradoxical increase in P1NP may reflect PTHrP recruitment of osteoblast precursors and initiation of osteoblast differentiation which is then followed by an arrest of osteoblast differentiation at the pre-osteoblast to osteoblast transition [31–33]. This hypothesis may also explain the rapid increase in BMD after weaning as the pre-osteoblasts are poised to rapidly complete their differentiation once PTHrP levels decreased. Though bone histomorphometric data would be helpful to validate this theory, it is not feasible to collect these samples during lactation.

Interestingly, six months after weaning, P1NP levels returned to baseline in the AA group but fell to below baseline in the C group which may partially explain the racial difference in recovery of FN BMD. The racial difference in P1NP post-weaning may be explained in part by the significantly higher estradiol levels measured in the AA group. Previous studies that show that estradiol levels are up to ~ 50% higher in AA women compared to C throughout the menstrual cycle [34]. We were not able to identify any studies comparing estradiol levels during lactation and weaning in AA vs C women. Although we were not able to control for timing in menstrual cycle when measuring estradiol levels, it seems unlikely that this would explain the differences between the groups.

Vitamin D levels were higher than we reported in previous studies, and although there was no difference in serum calcium, total daily calcium intake was also significantly higher than average in both groups which may reflect selection bias. Vitamin D intake was not quantified, which is a limitation of this study. Of the prenatal vitamins the participants were using, most contained 400 IU vitamin D3, but some contained up to 1000 IU. Participants were recruited throughout the year so there were no seasonal variations in timing of visits that could explain differences in vitamin D levels. Vitamin D decreased slightly in both groups compared to baseline, though in the AA group the decrease was statistically significant only at the 6 month visit. PTH levels were stable in both groups, indicating that the small changes in vitamin D were not clinically significant. 1,25 vitamin D levels as well as vitamin D binding protein were not measured which is a recognized limitation of the study.

Average PTH levels were within the reference range throughout the study and did not differ between groups. Although some have reported higher PTH levels in AA compared to C women, our study found similar average PTH levels. This may be because vitamin D levels were fairly robust in both groups and therefore above the threshold for PTH secretion in both [35]. It is of note that samples for PTH were frozen and run in a batch assay at the completion of the study. Therefore, there were individuals with elevated PTH levels at baseline (AA n = 4, C n = 3) who were included in the study. The study was not powered to determine the effect of baseline PTH on bone turnover or bone mineral density.

The small sample size, particularly in the AA group at the post-wean visit, and the presence of outliers in several of the measurements which may have affected the results, may limit the ability to detect small changes. Ideally a similar study would be done with a larger sample over a longer time interval.

An additional limitation of the study is that PTHrP levels were not measured. Thus, unanswered questions remain regarding whether the AA skeleton is equally responsive to PTHrP in the hormonal milieu of lactation. Since PTH and PTHrP bind to the same PTH1 receptor, it is possible that the total combined levels of PTH and PTHrP may have contributed to the observed changes in bone density and bone turnover. However, given the short half-life of PTHrP, it is unlikely that PTHrP levels would have an effect on bone turnover 6 months after ceasing lactation.

We were concerned about possible racial inequities that affected recruitment for our study may also reflect factors that impact bone health in the wider population. Despite the many proven benefits of breastfeeding, in the United States, AA women have a lower rate of initiation of breastfeeding and continuation than other races [36]. Many different cultural, sociological, health and other factors contribute to this difference in breastfeeding rates [37]. Many AA mothers were excluded from our study due to higher use of DMPA which was administered prior to discharge from the hospital for birth control compared to their C counterparts. While it can prevent pregnancy, DMPA is known to cause bone loss and has been associated with increased fracture risk [38]. Though bone density does appear to recover after discontinuation of medroxyprogesterone in the general population [39], further studies are needed to determine the effects of administering the medication during lactation.

In summary, despite differences in baseline BMD, there was a decrease in BMD in both AA and C new mothers during the first 6 months of lactation, with a slightly greater decrease for the C group at the TH and FN. By 6 months after weaning, BMD returned to baseline at all sites in the AA group, but had not completely recovered compared to the 2 week postpartum baseline at the FN in the C group. Changes in markers of bone formation and resorption were similar between groups during lactation. However, bone formation was significantly lower in the C group after weaning which may explain the lack of recovery of BMD at the FN. Further research needs to be done to determine if there are impacts on long term bone health for women who do not fully recover BMD before a subsequent pregnancy. Additionally, the effect of DMPA on bone metabolism when given to lactating women immediately postpartum requires further study in all racial groups.

## References

[1] Laskey MA, Prentice A, Shaw J, Zachou T, Ceesay SM, Vasquez-Velasquez L, Fraser DR (1990). Breast-milk calcium concentrations during prolonged lactation in British and rural Gambian mothers. Acta Paediatr Scand.

[2] Kovacs CS (2001). Calcium and bone metabolism in pregnancy and lactation. J Clin Endocrinol Metab.

[3] (2011) Institute of Medicine (US) Committee to review dietary reference intakes for vitamin D and calcium. In: Ross AC, Taylor CL, Yaktine AL et al (ed) 5, Dietary reference intakes for adequacy: calcium and vitamin D. National Academies Press (US), Washington (DC) 21796828

[4] Sowers M, Corton G, Shapiro B, Jannausch ML, Crutchfield M, Smith ML, Randolph JF, Hollis B (1993). Changes in bone density with lactation. JAMA.

[5] Teerapornpuntakit J, Chanprapaph P, Karoonuthaisiri N, Charoenphandhu N (2017). Site-specific onset of low bone density and correlation of bone turnover markers in exclusive breastfeeding mothers. Breastfeed Med.

[6] Drinkwater BL, Chesnut CH (1991). Bone density changes during pregnancy and lactation in active women: a longitudinal study. Bone Miner.

[7] More C, Bettembuk P, Bhattoa HP, Balogh A (2001). The effects of pregnancy and lactation on bone mineral density. Osteoporos Int.

[8] Kalkwarf HJ, Specker BL, Bianchi DC, Ranz J, Ho M (1997). The effect of calcium supplementation on bone density during lactation and after weaning. N Engl J Med.

[9] Brembeck P, Lorentzon M, Ohlsson C, Winkvist A, Augustin H (2015). Changes in cortical volumetric bone mineral density and thickness, and trabecular thickness in lactating women postpartum. J Clin Endocrinol Metab.

[10] Bjornerem A, Ghasem-Zadeh A, Wang X, Bui M, Walker SP, Zebaze R, Seeman E (2017). Irreversible deterioration of cortical and trabecular microstructure associated with breastfeeding. J Bone Miner Res.

[11] Carneiro RM, Prebehalla L, Tedesco MB, Sereika SM, Hugo M, Hollis BW, Gundberg CM, Stewart AF, Horwitz MJ (2010). Lactation and bone turnover: a conundrum of marked bone loss in the setting of coupled bone turnover. J Clin Endocrinol Metab.

[12] Cross NA, Hillman LS, Allen SH, Krause GF (1995). Changes in bone mineral density and markers of bone remodeling during lactation and postweaning in women consuming high amounts of calcium. J Bone Miner Res.

[13] Yamaga A, Taga M, Minaguchi H, Sato K (1996). Changes in bone mass as determined by ultrasound and biochemical markers of bone turnover during pregnancy and puerperium: a longitudinal study. J Clin Endocrinol Metab.

[14] Sowers M, Eyre D, Hollis BW, Randolph JF, Shapiro B, Jannausch ML, Crutchfield M (1995). Biochemical markers of bone turnover in lactating and nonlactating postpartum women. J Clin Endocrinol Metab.

[15] Sowers MF, Hollis BW, Shapiro B, Randolph J, Janney CA, Zhang D, Schork A, Crutchfield M, Stanczyk F, Russell-Aulet M (1996). Elevated parathyroid hormone-related peptide associated with lactation and bone density loss. JAMA.

[16] VanHouten JN, Wysolmerski JJ (2003). Low estrogen and high parathyroid hormone-related peptide levels contribute to accelerated bone resorption and bone loss in lactating mice. Endocrinology.

[17] Kovacs CS, Kronenberg HM (1997). Maternal-fetal calcium and bone metabolism during pregnancy, puerperium, and lactation. Endocr Rev.

[18] Pearson D, Kaur M, San P, Lawson N, Baker P, Hosking D (2004). Recovery of pregnancy mediated bone loss during lactation. Bone.

[19] Moller UK, Vieth Streym S, Mosekilde L, Rejnmark L (2012). Changes in bone mineral density and body composition during pregnancy and postpartum. A controlled cohort study. Osteoporos Int.

[20] Cooke-Hubley S, Kirby BJ, Valcour JE, Mugford G, Adachi JD, Kovacs CS (2017). Spine bone mineral density increases after 6 months of exclusive lactation, even in women who keep breastfeeding. Arch Osteoporos.

[21] Cosman F, Nieves J, Dempster D, Lindsay R (2007). Vitamin D economy in blacks. J Bone Miner Res.

[22] Powe CE, Karumanchi SA, Thadhani R (2014). Vitamin D-binding protein and vitamin D in blacks and whites. N Engl J Med.

[23] Cosman F, Morgan DC, Nieves JW, Shen V, Luckey MM, Dempster DW, Lindsay R, Parisien M (1997). Resistance to bone resorbing effects of PTH in black women. J Bone Miner Res.

[24] Ettinger B, Sidney S, Cummings SR, Libanati C, Bikle DD, Tekawa IS, Tolan K, Steiger P (1997). Racial differences in bone density between young adult black and white subjects persist after adjustment for anthropometric, lifestyle, and biochemical differences. J Clin Endocrinol Metab.

[25] Sawo Y, Jarjou LM, Goldberg GR, Laskey MA, Prentice A (2013). Bone mineral changes after lactation in Gambian women accustomed to a low calcium intake. Eur J Clin Nutr.

[26] Prentice A, Jarjou LM, Cole TJ, Stirling DM, Dibba B, Fairweather-Tait S (1995). Calcium requirements of lactating Gambian mothers: effects of a calcium supplement on breast-milk calcium concentration, maternal bone mineral content, and urinary calcium excretion. Am J Clin Nutr.

[27] Prentice A, Jarjou LM, Stirling DM, Buffenstein R, Fairweather-Tait S (1998). Biochemical markers of calcium and bone metabolism during 18 months of lactation in Gambian women accustomed to a low calcium intake and in those consuming a calcium supplement. J Clin Endocrinol Metab.

[28] Carneiro RM, Prebehalla L, Tedesco MB, Sereika SM, Gundberg CM, Stewart AF, Horwitz MJ (2013). Evaluation of markers of bone turnover during lactation in African-Americans: a comparison with Caucasian lactation. J Clin Endocrinol Metab.

[29] Payne RB (1998). Renal tubular reabsorption of phosphate (TmP/GFR): indications and interpretation. Ann Clin Biochem.

[30] Grizzo FMF, Alarcao ACJ, Dell' Agnolo CM, Pedroso RB, Santos TS, Vissoci JRN, Pinheiro MM, Carvalho MDB, Pelloso SM (2020). How does women's bone health recover after lactation? A systematic review and meta-analysis. Osteoporos Int.

[31] van der Horst G, Farih-Sips H, Lowik CW, Karperien M (2005). Multiple mechanisms are involved in inhibition of osteoblast differentiation by PTHrP and PTH in KS483 Cells. J Bone Miner Res.

[32] Wang YH, Liu Y, Buhl K, Rowe DW (2005). Comparison of the action of transient and continuous PTH on primary osteoblast cultures expressing differentiation stage-specific GFP. J Bone Miner Res.

[33] Dobnig H, Turner RT (1997). The effects of programmed administration of human parathyroid hormone fragment (1–34) on bone histomorphometry and serum chemistry in rats. Endocrinology.

[34] Marsh EE, Shaw ND, Klingman KM, Tiamfook-Morgan TO, Yialamas MA, Sluss PM, Hall JE (2011). Estrogen levels are higher across the menstrual cycle in African-American women compared with Caucasian women. J Clin Endocrinol Metab.

[35] Aloia JF, Chen DG, Chen H (2010). The 25(OH)D/PTH threshold in black women. J Clin Endocrinol Metab.

[36] Beauregard JL, Hamner HC, Chen J, Avila-Rodriguez W, Elam-Evans LD, Perrine CG (2019) Racial Disparities in Breastfeeding Initiation and Duration Among U.S. Infants Born in 2015. MMWR Morb Mortal Wkly Rep 68:745–748. 10.15585/mmwr.mm6834a3 10.15585/mmwr.mm6834a3 PMC6715261 31465319

[37] Gyamfi A, O'Neill B, Henderson WA, Lucas R (2021). Black/African American breastfeeding experience: cultural, sociological, and health dimensions through an equity lens. Breastfeed Med.

[38] Meier C, Brauchli YB, Jick SS, Kraenzlin ME, Meier CR (2010). Use of depot medroxyprogesterone acetate and fracture risk. J Clin Endocrinol Metab.

[39] Kaunitz AM, Arias R, McClung M (2008). Bone density recovery after depot medroxyprogesterone acetate injectable contraception use. Contraception.

